# The Association between Coparenting Behavior and Internalizing/Externalizing Problems of Children and Adolescents: A Meta-Analysis

**DOI:** 10.3390/ijerph191610346

**Published:** 2022-08-19

**Authors:** Fengqing Zhao, Haomeng Wu, Yixuan Li, Huifang Zhang, Jie Hou

**Affiliations:** 1 School of Education, Zhengzhou University, Zhengzhou 450001, China; 2 Department of Psychology, Renmin University of China, Beijing 100872, China

**Keywords:** coparenting, internalizing problems, externalizing problems, meta-analysis

## Abstract

This study aimed to determine the association between coparenting behavior and children’s externalizing and internalizing problems and possible factors that may moderate their associations. A meta-analysis of 93 studies involving 41,207 participants found that coparenting behavior was slightly and significantly related to externalizing problems, r = −0.17, 95% CI [−0.194, −0.15], and internalizing problems, r = −0.16, 95% CI [−0.18, −0.14]. In addition, coparenting integrity, cooperation, conflict, competitiveness, and triangulation were significantly related to externalizing and internalizing problems. Moderation analyses revealed the following findings: (a) data reporter moderated the association between coparenting and internalizing problems, with children-report coparenting showing a significantly stronger relation with internalizing symptom than father-report coparenting; (b) developmental stage was found to moderate the association between coparenting behavior and externalizing problems, with stronger association found in childhood than in toddlerhood; (c) female percentage, individualism–collectivism culture, research methods, and publication year were not found to moderate the association between coparenting behavior and externalizing or internalizing problems. These findings help summarize the previous studies and provide an empirical basis for the relation between coparenting and child externalizing/internalizing problems, and benefits targeted interventions towards coparenting behaviors.

## 1. Introduction

Parental cooperation and sharing responsibilities in childrearing play an important role in children’s healthy development [1]. Many empirical studies have aimed to explore coparenting behavior and its influence on children’s mental health in recent decades. Results have shown that positive coparenting is beneficial to child positive adjustment, and negative coparenting leads to a series of internalizing and externalizing problems in later life [2,3,4,5,6,7]. However, empirical evidence on the size of the relation between coparenting and internalizing and externalizing problems is mixed [8], with some studies indicating a low to moderate relation (|r| < 0.30; e.g., [2,9]), other studies reporting a high relation (|r| = 0.62; e.g., [9]), and yet further studies showing a nonsignificant relation [5,10]. With respect to the dimensions of coparenting, some controversies still exist regarding the categorization of coparenting behavior, as well as the relationship between coparenting dimensions and child adjustment problems [11]. In addition, since these studies were based on relatively small sample size, it is necessary to conduct a meta-analysis to gain a better understanding about the degree to which coparenting is related to internalizing and externalizing problems, the dimension that contribute more to children’s mental health problems, and how the study characteristics influence the effect-size estimates and cause the heterogeneity of results.

### 1.1. Coparenting Behavior

Coparenting behavior refers to the collaboration in childrearing of two parental figures who share responsibilities for at least one child, and emphasizes how parents work with or against each other when caring for their child [12]. Family system theory proposes that family is a complex consisting of a series of subsystems, including the marital subsystem, parent–child subsystem, and coparenting subsystem [13]. Based on this theory, coparenting is a distinct concept relating to parental interplay in childrearing [8]. Farr and Patterson further affirmed that coparenting serves as “the family’s executive subsystem” [3]. It may independently influence the development of individuals within the family [14] or interact with other subsystems to affect family members’ functioning and well-being [15,16,17].

With more and more studies concerning coparenting emerging in recent years, researchers have put forward diverse categorizations of the dimensions and measurements of coparenting. Therefore, an overview of these studies is necessary to enable meta-analysis to be conducted using consistent terms. Originally, McHale used the observation method to categorize coparenting into three factors, namely, hostility–competitiveness [1], family harmony, and parenting discrepancy. Later, McHale amended the three factors of coparenting to affective contact, support/mutuality, and coparental conflict [18]. McHale further distinguished parents’ own overt and covert activities and categorized coparenting into family integrity, conflict, disparagement, and reprimand [19]. Similarly, McConnell et al. [20] used McHale’s [1] observation method and divided coparenting into three dimensions: family harmony, parenting discrepancy, and hostility–competitiveness. Feinberg describes coparenting as containing four components: childrearing agreement, division of labor, support/undermining, and joint family management [12]. Murphy considered that coparenting is comprised of competitiveness, cooperation, family conflict, and negative affect [21]. Though these studies focus on different aspects and use different terms to define coparenting behavior, they generally consider that family harmony, affective contact, and family integrity serve as important indicators of family cohesion, as they all describe the overall regularity of warm, engaging interactions among family members. Support/mutuality has been used to describe parents’ mutual support for one another and mutual involvement with the child. Coparental conflict, disparagement, discrepancy, and reprimand all serve as indicators to describe coparenting teams showing various levels of overt antagonism. Hostility–competitiveness emphasizes the competitiveness among the family triad and is an indicator that describes parent-centered, negativistic one-upmanship in the coparenting relationship [18,19].

To sum up, previous studies almost reach an agreement that coparenting includes both positive and negative dimensions [2,3,4,5,6,7,22,23]. Positive coparenting includes cooperation and family integrity, whereas negative coparenting includes conflict, competitiveness and triangulation.

Cooperative coparenting is defined as the extent to which partners work together as a team, show active support for one another’s interventions with the child, and communicate to the child with a climate of mutual loyalty [3,7,10,11,22,24,25,26]. Therefore, studies using the terms “support”, “cooperation”, and “communication” to emphasize the collaborative behavior between father and mother about childrearing were regarded as coparenting cooperation in this article. Furthermore, the term “coparenting agreement” used in different articles, stressing that parental figures reach an agreement on a range of child-related topics, was also considered as an aspect of coparenting cooperation [12]. For example, in an observation of the clothes changing task, cooperation behavior was coded when parents show responsivity to each other, such as asking and answering questions, agreeing, and going along with the partner’s initiatives [10]. In addition, other terms including “coparenting banter” [27] and “shared decision making” [28] were also categorized as coparental cooperation.

Integrity refers to the degree to which coparenting behavior servers to promote harmony, warmth, and cohesiveness among family members [1,9,27,29], and serves as an important indicator of family cohesion. Terms such as “family harmony”, “affective contact”, and “family integrity” were all categorized into this dimension. The big difference between cooperation and integrity in this paper is that the former focuses on interparental interaction about childrearing, but integrity concentrates on triadic relations, especially the general affect among family members.

Coparental conflict is defined as the extent of parental verbal sparring, hostile and critical remarks in the presence of their child, and disagreements in childrearing [11,30,31,32]. Specifically, there are two kinds of coparenting conflict: the first is the disagreement, conflict, and inconsistency over the childrearing problem; and the second is the conflict, hostility, and withdrawal between the father and mother that is perceived by children.

Coparental competitiveness–triangulation is an integration of competitiveness [10,11] and triangulation [31,33,34,35,36,37,38,39,40,41] to describe the dilemma that children have to take the side of either father or mother. Triangulation refers to the situation in which children often feel caught in the middle or torn between parents [42]. For instance, mothers ask their children to take a side with one of their parents through some utterance and behavior after a fight with their partners. Competitive coparenting has a similar meaning to triangulation in some studies, and was defined as one parent undermining the other in the presence of the child or jockeying for control of the child, and has been identified as a robust predictor of externalizing symptoms in children [11]. Both triangulation and competition focus on the triadic relationship [1,11,21,34]. Therefore, it is reasonable to put triangulation and competition under the same heading and differentiate them from conflictual coparenting.

### 1.2. Coparenting and Internalizing/Externalizing Problems

Previous studies have documented that coparenting quality is linked to individuals’ mental health problems [29,43], from toddler period [32,44] and childhood [11] to adolescence [30]. Specifically, some cross-sectional findings have confirmed that positive coparenting was negatively associated with internalizing/externalizing problems [45,46]. In contrast, children living in a negative coparenting context would have more internalizing/externalizing problems [3,6]. In addition, longitudinal studies indicate that a supportive coparenting context in the infant period can predict lower levels of dysregulation and externalizing behavior after 18 months [2], and fewer child behavioral problems after 6 years in school children [22]. In contrast, undermining coparenting was positively associated with externalizing symptom after 1 year [7], and affective disorder and somatic complaints after 5 years [26]. Furthermore, coparenting can independently contribute to the child behavior outcomes even after controlling for individual parenting styles such as fathers’ sensitive guidance [29], intrusiveness of parenting [47], parenting warmth [48], and individual hash parenting [26].

Coparenting dimensions may contribute to child adjustment problems in different ways. First, both coparenting cooperation and family integrity have been negatively associated with child adjustment problems. For instance, coparenting cooperation negatively predicted internalizing/externalizing problems [49], and positively predicted children’s prosocial behavior after 6 months [25]. Similarly, coparenting integrity was negatively related to internalizing/externalizing symptoms such as conduct problems, anxiety, sadness, and depression [19,27,50,51]. Second, coparenting conflict as a risk factor was positively associated with a series of externalizing symptoms such as risk behaviors [24], ADHD [52], ODD [26], and internalizing problems [53] such as anxiety [54], depression [30], and somatic complaints [26]. Finally, both competitive coparenting and triangulation were associated with the increase in internalizing and externalizing problems [11,26,33,39].

However, the existing findings about the relationship between coparenting and child adjustment problems have not reached an agreement. Though many studies indicate that undermining coparenting contributes to more externalizing and internalizing problems, a nonsignificant association was found in both cross-sectional studies [3] and longitudinal studies [7]. With respect to the dimension of coparenting, some controversies were found in triangulation and conflict. For example, some studies found a nonsignificant association between triangulation and children’s depression in both cross-sectional studies [41] and longitudinal studies [24]; in contrast, other studies indicated that triangulation was significantly associated with internalizing problems but not significantly related to externalizing problems in children aged 10 to 12 years old [39]. A similar controversy exists in studies related to the association between coparenting conflict and internalizing and externalizing problems, with the association ranging from non-significant [55] to significantly positive [24,49]. These controversies provide a valuable understanding of the relation between coparenting and children’s adjustment problems; however, these existing controversies indicate that some study characteristics (e.g., developmental stage, gender, and data reporter) may influence the relation between coparenting and adjustment problems and cause the heterogeneity of results. Therefore, a meta-analysis is needed to classify this inconsistency and discover the potential study characteristics that moderate the relation between coparenting and child adjustment problem.

### 1.3. Development Stage

Age or developmental stage is an important factor in understanding the relationship between coparenting and internalizing/externalizing problems, which can be conceptualized within two different standpoints. The first perspective holds that the association between coparenting and adjustment problems is stronger in younger children than older children. Younger children are highly dependent on parents and spend more time with parents, whereas those who are in the adolescence period struggle to escape from parents’ monitoring, and spend less time at home and more time at school with peers. Thus, both children’s perceived coparenting and the influence of coparenting on children’s adjustment problems may decrease with age. For instance, age was negatively related to coparenting cooperation in a sample of adolescents (r = −0.11) [24], whereas the association between coparenting and age was not significant in a sample of preschoolers (r = −0.003) [29]. The second perspective holds that coparenting may influence internalizing and externalizing behavior in different ways across different age stages. A previous study reported a rise in internalizing symptoms such as depression with increase in age [8]. For example, an investigation among children from 3 years old to 18 years old indicates that age was negatively associated with externalizing symptoms (r = −0.12) and positively associated with internalizing symptoms (r = 0.24) [49]. In addition, a meta-analysis found a smaller effect size in the association between coparenting and internalizing symptoms, and a non-significant association between coparenting and externalizing symptoms with the increase in age [8]. Based on these two standpoints, it is necessary to examine the moderating effect of developmental stage on the association between coparenting and externalizing/internalizing symptoms.

### 1.4. Gender Difference

Regarding gender difference, there are two different explanatory models: the male vulnerability model [56,57] and the distinct pathways model [58,59]. The distinct pathways model contends that boys and girls may be differentially affected by stressful life events such that boys perform more externalizing problems and girls have more internalizing problems when facing stressors. Some cross-sectional studies [18,60] and longitudinal studies [26] have confirmed a differential reactivity model in which boys have more externalizing symptoms, whereas girls living in a negative coparenting context are more likely to show internalizing symptoms. The male vulnerability model claims that boys are more vulnerable to negative coparenting [59,61]. Thus, compared to girls, boys show more internalizing or externalizing problems when they live in a negative coparenting context. Thus, there is a need to examine the moderating effect of gender in the association between coparenting and externalizing/internalizing problems.

### 1.5. Data Reporter

Another important factor influencing the relationship between coparenting and children’s adjustment problems is the data reporter [62,63]. Previous studies indicate that father-report and mother-report coparenting have a significant correlation (e.g., r = 0.34~0.63) [45,64], especially in terms of coparenting conflict [30,65]. However, some studies indicate that father-report coparenting has a stronger relation with children’s adjustment problems (r = −0.35) than mother-report coparenting (r = −0.04) [41]. In addition, compared to father-report and mother-report coparenting, children’s perception of coparenting quality may be a more proximal factor influencing their adjustment problems. Generally, the quality of coparental alliance may influence children’s perception of coparenting and, in turn, influence children’s closeness to and satisfaction with both parents [66,67]. This evidence indicates that child-report coparenting might be different from father- or mother-report coparenting. Therefore, it is necessary to examine whether father-report, mother-report, and child-report coparenting contribute differently to children’s internalizing/externalizing problems.

### 1.6. Current Study

A previous meta-analytic review examined the effect of coparenting on child behavioral outcomes and some potential moderators such as age of children, percentage of girls, and percentage of separated parents [8]. Although this study provided a valuable insight into understanding the relation between coparenting and child outcomes, the results should be interpreted cautiously due to several limitations. First, this study was conducted more than ten years ago, and more studies emerged in recent years. It is necessary to conduct a new meta-analysis to cover recent studies. Second, important factors related to study characteristics that may influence the relation between coparenting and child adjustment were not considered. To date, few studies have systematically examined the association between the four dimensions of coparenting (cooperation, integrity, conflict, and competitiveness–triangulation) and child internalizing and externalizing problems [23]. Therefore, in the current meta-analysis, we aimed to quantitatively examine the association between the four coparenting dimensions and child internalizing/externalizing problems, and then focus on potential moderators. We anticipate that this meta-analysis will address the following three research aims:

1. Provide an overview of the relation between coparenting and child adjustment problems and quantify the overall effect of the association.
2. Explore different effect sizes between four coparenting dimensions and child externalizing/internalizing problems in cross-sectional studies and longitudinal studies.
3. Determine how the study characteristics moderate the effects of coparenting on child externalizing/internalizing problems.

## 2. Method

### 2.1. Search for Studies and Inclusion Criteria

Articles for this meta-analysis were identified from three main sources. First, the primary source for studies was a comprehensive literature review of articles listed in five academic databases (ISI Web of Knowledge, PsycINFO, PsycArticles, CNKI, and Google Scholar) that were published before 1 June 2019. Relevant publications were systematically searched with the following two groups of keywords: (1) (coparenting OR co-parenting OR parenting alliance OR triangulation OR family system OR parental agreement OR child-related disagreement OR marital discord over childrearing OR parental agreement regarding child-rearing OR interparental conflict OR coparental conflict OR coparental agreement OR coparental disagreement OR interparental disagreement OR coparental cooperation OR interparental cooperation OR competitive coparenting); (2) (adolescent OR youth OR childhood OR toddlerhood OR school-aged). Second, the studies used in the previously published meta-analysis [8] were considered for inclusion. Third, we searched in previous relevant reviews and also contacted researchers to request correlation tables that were not provided in their reported studies. These three methods initially yielded a total of 2023 studies.

### 2.2. Inclusion and Exclusion Criteria

**Figure 1:**
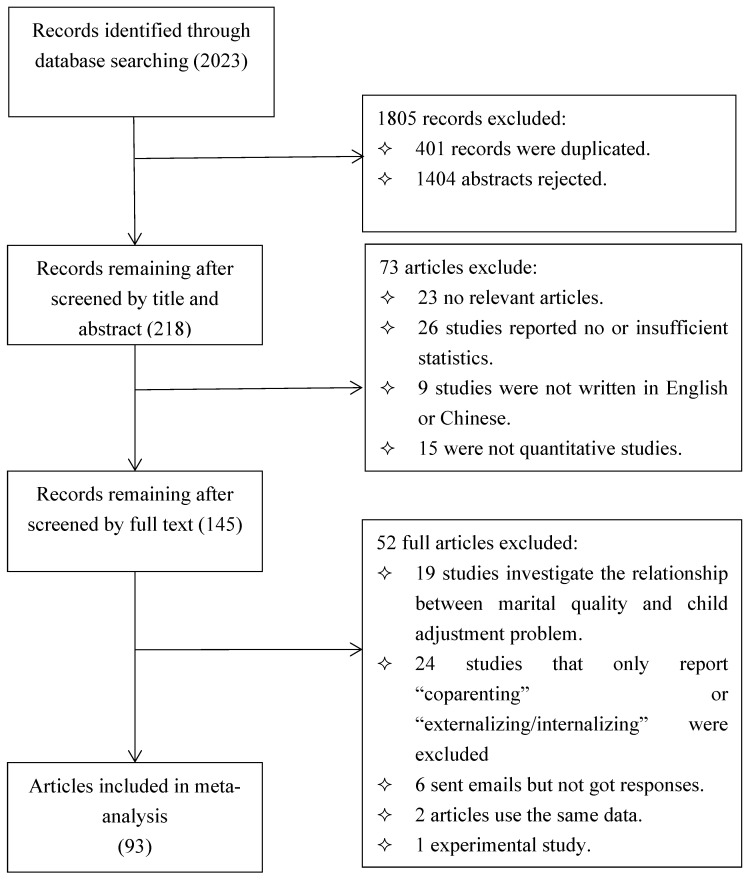
The PRISMA flowchart for the studies included in systematic review and meta-analysis.

Our search resulted in 2023 records for screening to identify eligible studies. Three authors of this study then examined the titles and abstracts of all the references and discarded irrelevant ones. After excluding the duplicated 401 articles and 1404 irrelevant articles, the remaining 218 articles were included in or excluded from our meta-analysis based on the following criteria: (a) The studies needed to be empirical and quantitative. (b) Pearson correlation coefficients were provided; otherwise, sufficient information from which an effect size could be derived needed to be available; specifically, if the correlation coefficients were not reported in the study, but the statistics of t, χ2 and F were reported, the corresponding formula [r = $\sqrt{\frac{t2}{t2\;+\;\mathrm{df}}}$; r = $\sqrt{\frac{\chi 2}{\chi 2\;+\;N}}$; r = $\sqrt{\frac{F}{F\;+\;\mathrm{dfe}}}$; r = β × 0.98 + 0.05 (β ≥ 0); r = β × 0.98 − 0.05 (β < 0) (β ∈ (−0.5, 0.5))] was used to convert these statistics into r [68]. (c) Studies had to be published in English or Chinese. (d) Studies that did not focus on the coparenting relationship but rather on the romantic, sexual, and marital relationship between two parental figures were excluded. (e) Data indicating that only one parent engaged in coparenting behavior in an observation situation was regarded as parenting behavior and excluded. (f) Studies that only reported the indices of “coparenting” or “externalizing/internalizing” were excluded. (g) Studies violating the assumption of independent samples (e.g., multiple effect sizes were obtained from one independent sample and could not be integrated into one effect size) were excluded. Finally, 93 studies met the criteria for inclusion. The PRISMA flow diagram is depicted in Figure 1.

### 2.3. Coding

Studies that met the inclusion criteria were coded for sample characteristics (for details refer to Table S1 in Supplementary Materials). The first author developed a coding manual that specified the coding categories and possible codes to be used for each study. We entered the following variables: name of the authors, year of publication, ethnicity of the family, country, mono-informant bias in the data (dummy coding: yes = 1 vs. no = 0), annual family income, marital length, percentage of separated parents, sibling, longitudinal research design (dummy coding: yes = 1 vs. no = 0), the number of participants (separately for children, mothers and fathers), mean age of participants (separately for children, mothers and fathers), mean age of participants (separately for children, mothers and fathers), percentage of father and mother completed high school, and the percentage of girls. In addition, we coded four coparenting dimensions (cooperation, integrity, conflict, and triangulation), in addition to assessment methods of coparenting (questionnaire, indirect assessment, observation, and interview), and child adjustment categories (internalizing problems, externalizing problems), assessment methods (questionnaire and observation), and the number of reporters. Following the coding manual, the authors coded all information contained in the 93 studies. Two authors independently coded each report, and any discrepancies were resolved by consensus, bringing in the third author if necessary. For controversial studies, the three authors fully discussed whether to include them according to the definition of coparenting and internalizing/externalizing problems. Agreement was reached in 96% of the cases using two coders’ coding, which indicated that the literature coding of this study was accurate and effective.

### 2.4. Statistical Method

The effect size index used for all outcome measures is Pearson’s r, indicating the correlation between coparenting and internalizing/externalizing symptoms. If the Pearson correlation was not available, we accepted alternative statistics such as F, T, and χ2. Analysis was based on the recommendations of Borenstein et al. [69]. Specifically, we converted the correlation coefficient to Fisher’s Z scale, and all analyses were performed using the transformed values. The results, such as the summary effect size and its confidence interval, were converted back to a raw correlation for presentation. In addition, random-effects meta-regression models were run in R to summarize effect size and to examine potential moderators [70]. A random-effects model assumes the observed estimates of the association between coparenting and externalizing/internalizing problems can vary across studies because real differences lie in the study characteristics and other factors, in addition to the sampling variability.

First, we meta-analyzed the magnitude of the effect size between coparenting and internalizing/externalizing problems. Then, subgroup analyses were used to examine the relations between coparenting dimensions and externalizing/internalizing problems for each moderator. Meta-regression analyses were used to examine whether types of coparenting, age, percentage of girls, research method (cross-sectional or longitudinal), data reporter, and mono-informant bias moderated the relation between coparenting and internalizing/externalizing symptoms. For the moderation analysis, all moderators were entered into the model simultaneously. With respect to categorical moderators, we created dummy coded variables to examine the comparisons among categories [71]. Finally, publication bias was examined using the funnel plot and Egger’s test [72].

## 3. Results

Based on our inclusion criteria, 93 studies (including 2 unpublished articles; 60 cross-sectional studies and 33 longitudinal studies) involving 125 effect sizes, 41,207 participants, and 612 correlation coefficients were included for the final analyses. The sample sizes ranged from 21 to 9050 participants. Children’s average age ranged from 2 to 18.91 years. A total of 82 studies reported the children’s gender ratio, among which the proportion of girls ranged from 23% to 79%. Twenty-five studies indicated that their data came from the same reporter, indicating possible mono-informant bias. Regarding ethics, we used Hofstede’s cultural dimensions theory to calculate and analyze individualism/collectivism according to ethnic compositions, and the proportion of individualism ranged from 20% to 91%.

The correlation between coparenting and child adjustment problems was r = −0.182, 95%CI = [−0.208, −0.156], and the effect size between coparenting and externalizing problems was r = −0.173, 95%CI = [−0.194, −0.151], and that between coparenting and internalizing problems was r = −0.162, 95% CI = [−0.180, −0.144]. More detailed information is shown in Table 1.

### 3.1. Coparenting Dimensions and Externalizing/Internalizing Problems

#### 3.1.1. Coparenting Dimensions and Externalizing/Internalizing Problems in Cross-Sectional Studies

With respect to the relations between coparenting and externalizing problems, there were 20 independent samples (38 correlations) involving integrity, 34 independent samples (55 correlations) involving cooperation, 67 independent samples (158 correlations) involving interparental conflict, and 15 independent samples (33 correlations) involving C-T. As Table 1 shows, the average correlation between externalizing problems and coparenting for each of the four coparenting dimensions was significant: integrity, r = −0.141, 95%CI = [−0.252, −0.028]; cooperation, r = −0.111, 95%CI = [−0.153, −0.068]; conflict, r = 0.201, 95%CI = [−0.231, −0.171]; C-T, r = 0.168, 95%CI = [−0.221, −0.113]. These four effect sizes had slightly significant differences (Q = 12.669, *p* = 0.013). Specifically, as Table 2 shows, integrity was significantly more strongly related to externalizing problems compared to cooperation (β = 0.228, t = 0.078, 95%CI = [0.074, 0.382]), conflict (β = 0.255, t = 0.070, 95%CI = [0.117, 0.393]), and C-T (β = 0.179, t = 0.080, 95%CI = [0.337, 0.023]). No significant differences were found in other pairwise comparisons.

With respect to the relations between coparenting and internalizing symptom, there were 19 independent samples (35 correlations) involving integrity, 30 independent samples (53 correlations) involving cooperation, 74 independent samples (187 correlations) involving conflict, and 16 independent samples (33 correlations) involving C-T. As Table 1 shows, the average correlation between internalizing problems and coparenting for each of the coparenting dimensions was significant: integrity, r = −0.153, 95%CI = [−0.202, −0.103]; cooperation, r = −0.121, 95%CI = [−0.158, −0.084]; conflict, r = 0.189, 95%CI = [−0.215, −0.163]; C-T, r = 0.126, 95%CI = [−0.158, −0.065]. As Table 2 shows, after controlling for covariates and other moderators, no significant differences were found in comparison.

#### 3.1.2. Coparenting Dimensions and Externalizing/Internalizing Problems in Longitudinal Studies

Next, we examined the effect size of correlation between coparenting and internalizing/externalizing symptoms from the longitudinal perspective. We extracted data of coparenting at the first time point and internalizing/externalizing at the last time point. With respect to longitudinal correlations between coparenting and externalizing symptom, there were 33 studies involving 65 correlations and the prediction time interval spanning from 0.3 to 14.5 years. As is shown in Table 3, Results showed that coparenting significantly predicted later externalizing problems, r = −0.144, 95%CI = [−0.173, −0.114], k = 65. Specifically, integrity significantly negatively predicted the later externalizing problems, r = −0.129, 95%CI = [−0.221, −0.035], k = 7; cooperation significantly negatively predicted the later externalizing problems, r = −0.076, 95%CI = [−0.115, −0.037], k = 18; conflict significantly positively predicted the later externalizing problems, r = 0.173, 95%CI = [−0.197, −0.150], k = 29; C-T significantly positively predicted the later externalizing problems, r = 0.122, 95%CI = [−0.161, −0.084], k = 9.

With respect to longitudinal correlations between coparenting and internalizing problems, there were 27 independent studies involving 56 correlations and the prediction time interval spanning from 0.3 to 14.5 years. Results showed that coparenting significantly predicted later internalizing problems, r = −0.125, 95%CI = [−0.153, −0.197]. Specifically, integrity significantly negatively predicted the later internalizing problems, r = −0.121, 95%CI = [−0.220, −0.019], k = 6; cooperation significantly negatively predicted the later internalizing problems, r = −0.069, 95%CI = [−0.116, −0.021], k = 13; conflict significantly positively predicted the later internalizing problems, r = 0.132, 95%CI = [−0.172, −0.091], k = 28; C-T significantly positively predicted the later internalizing problems, r = 0.135, 95%CI = [−0.176, −0.093], k = 7.

### 3.2. Moderation of Study Characteristics

#### 3.2.1. Moderation of Data Reporter

Moderation analyses revealed that the correlation between coparenting and externalizing problems were negative among father reporters (r = −0.179, 95%CI = [−0.239, −0.118]), mother reporters (r = −0.170, 95%CI = [−0.219, −0.121]), and child reporters (r = −0.179, 95%CI = [−0.225, −0.132]). These three effect sizes had no significant differences (Q = 0.239). As Table 2 shows, after controlling for covariates and other moderators, no significant differences were found in the data reporter comparison.

For the relation between coparenting and internalizing problems, father report: r = −0.117, 95%CI = [−0.172, −0.162]; mother report: r = −0.180, 95%CI = [−0.221, −0.138]; and child report: r = −0.232, 95%CI = [−0.265, −0.198]. These three effect sizes had significant differences (Q = 35.595, *p* < 0.001). As Table 2 shows, after controlling for covariates and other moderators, child-report coparenting was more strongly related to internalizing symptoms than father-report coparenting or mother-report coparenting.

#### 3.2.2. Moderation of Gender

As Table 2 shows, after controlling for covariates and other moderators, female percentage did not significantly influence the association between coparenting and internalizing, β = 0.002, t = 0.002, 95%CI = [−0.002, 0.005]; after controlling for covariates and other moderators, female percentage also did not significantly moderate the association of coparenting and externalizing: β < −0.01, t = 0.002, 95%CI = [−0.003,0.003].

#### 3.2.3. Moderation of Developmental Stage

We then examined the moderating effect of three developmental stages: toddler, child, and adolescent. With respect to coparenting and externalizing symptoms, 46 effect sizes were from studies based on participants in the toddler period, 58 effect sizes from studies based on participants in the child period, and 23 effect sizes from studies based on participants in the adolescent period. As Table 1 shows, the average correlation between coparenting and externalizing symptoms for each of the three developmental stage was significant: toddler, r = −0.134, 95%CI = [−0.177, −0.089]; child, r = −0.198, 95%CI = [−0.225, −0.163]; adolescent, r = −0.174, 95%CI = −0.221, −0.127, and Q = 6.458, *p* = 0.091. Table 2 further shows that after controlling for covariates and other moderators, no significant difference was found in development stage comparison.

With respect to coparenting and internalizing symptoms, 41 effect sizes were about the toddler period, 54 effect sizes about the child period, and 29 effect sizes were about the adolescent period. As Table 1 shows, the average correlation between coparenting and internalizing problems for each of the three developmental stages were significant: toddler, r = −0.164, 95%CI = [−0.201, −0.127]; child, r = −0.149, 95%CI = [−0.180, −0.117]; adolescent, r = −0.194, 95%CI = [−0.238, −0.281], and Q = 2.592, *p* = 0.459, such that the relation did not show significant differences between these three periods. As Table 2 shows, after controlling for covariates and other moderators, the relation between coparenting and internalizing symptoms was significantly larger in the child period than in the toddler or adolescent periods.

### 3.3. Publication Bias Analysis

**Figure 2:**
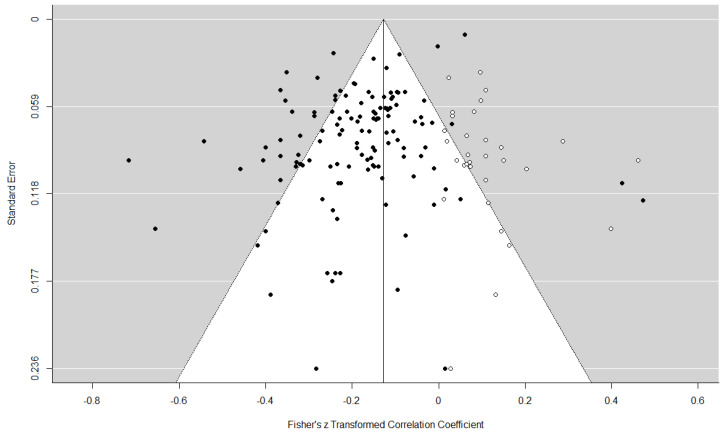
Funnel plot of publication bias. The closed dots indicate the observed studies, and the open dots indicate the missing studies imputed by trim-and-fill method (based on the estimator *L*_0_).

Since publication bias may affect the meta-analytic results and potentially produce overstated conclusions, we used a funnel plot to detect the publication bias, and used the trim-and-fill procedure to eliminate the publication bias. The funnel plot is a graphical tool used preliminarily to investigate publication bias. In order to achieve a more accurate evaluation, Egger’s regression method, which tests for a linear association between the effect estimates and their standard error, is used to test for funnel plot asymmetry [72]. As the funnel plot in Figure 2 shows, the research literature on the relationship between coparenting and child adjustment problems was evenly distributed on both sides of the total effective dose, which indicates that there may be no publication bias in the current research. The Egger’s test also did not find significant publication bias, with *p* = 0.093. Furthermore, we used the trim-and-fill procedure [73] to adjust for the numbers of the publication bias and the funnel plot; the results still confirmed that the effect between coparenting and child adjustment was statistically significant (r = −0.127, 95%CI = [−0.155, −0.100]).

## 4. Discussion

In the current meta-analysis, we investigated the relation between coparenting and children’s internalizing and externalizing problems, and whether study characteristics including development stage, gender, and data reporter influenced this relation. We also coded and examined possible moderation effects of other study characteristics such as mono-informant bias, research method, individualism, and female percentage.

### 4.1. Coparenting Dimensions and Externalizing/Internalizing Problems

The results first indicated that coparenting had a small but significant effect on children’s internalizing/externalizing problems, with the effect sizes ranging from −0.162 to −0.182. Specifically, both coparenting cooperation and integrity may decrease externalizing problems, whereas coparental conflict, competitiveness, and triangulation increase externalizing problems. Furthermore, both coparenting cooperation and integrity negatively predict internalizing problems, whereas coparenting conflict, competitiveness and triangulation positively predict internalizing problems. In addition, the pairwise comparisons further indicate that coparental conflict seems to be the most robust predictor of children’s externalizing and internalizing problems compared to the other coparenting dimensions, whereas coparental cooperation has the weakest significant association with externalizing/internalizing problems.

This finding is in line with previous studies [24,26,74]. Based on the results of coparenting behavior from different data reporters, father-, mother-, and child-reported coparenting show higher levels of consistency in the conflict dimension than other dimensions. This indicates that all family members are more sensitive to coparenting conflict. This might be because coparenting conflict is characterized by blatant, overt negative coparenting behavior compared with competitiveness and triangulation, and it is not easy to be neglected in the family environment. Thus, coparenting conflict shows high consistency among family members and results in the utmost influence on children’s adjustment problems. We encourage parents or adults to pay attention to the harm that coparenting conflict has on children’s psychological well-being.

In addition, in the negative coparenting context, the association between coparenting conflict and internalizing problems was stronger than the association between C-T and internalizing problems in cross-sectional studies. However, in longitudinal studies, conflict and C-T had a similar influence on child internalizing problems. Therefore, it is difficult to conclude whether coparenting conflict contributes more than C-T to children’s internalizing problems. However, based on the previous meta-analysis, triangulation may have a stronger relation with internalizing symptom than coparenting conflict [8]. This may be because triangulation is characterized as a rather covert inner family process, whereas coparenting conflict is understood as a more overt way for parents to act out their disagreements in childrearing [75]. Children exposed to covert processes such as triangulation may feel obliged to take one parent’s side, or may be afraid of their parents’ separation. Such feelings may manifest in internalizing symptoms rather than externalizing symptoms [35,75]. However, in our meta-analysis, the number of independent longitudinal studies regarding C-T is relatively small (k = 7), whereas the number or independent longitudinal studies about conflict was 28; therefore, possible biases may exist in the results. In addition, the number of studies related to the influence of overt and covert family conflict on children’s internalizing problems is also small. Therefore, we may cautiously conclude that both coparenting conflict and C-T have a significantly negative effect on child adjustment problems.

### 4.2. Moderation of Developmental Stage

A child’s developmental stage is an important factor influencing the relations between coparenting and externalizing/internalizing problems. To systematically examine the moderating effects of a child’s developmental stage, we included three developmental stage variables (infant, child, and adolescent). Our findings suggest that the relation between coparenting and externalizing problems showed a stronger correlation among children and adolescents than toddlers, whereas the association did not show a significant difference between children and adolescents. The finding is inconsistent with the previous meta-analysis, which found that children’s age did not emerge as a significant moderator [8]. Because parents’ coparenting is relatively consistent across age groups, the stronger association between coparenting and externalizing problems in childhood and adolescence compared to toddlers indicates that coparenting may have a delayed effect on children’s behavioral outcomes. In addition, when children enter the childhood period, they may begin to be involved in interparental conflict, and experience more negative emotions when experiencing coparenting triangulation and competitiveness. These negative perceptions may have a greater influence on children’s behavioral problems. Thus, parents, educators, and other practitioners should pay more attention to coparenting behavior in childhood and adolescence periods, and targeted interventions are encouraged to improve positive coparenting and decrease negative coparenting behaviors.

### 4.3. Moderation of Data Reporter

We found a significant association between coparenting and child externalizing symptoms in mother-report, father-report, and child-report data. Furthermore, the association did not show a significant statistical difference among these three kinds of data reporters. This result is consistent with previous studies indicating that father-report and mother-report coparenting are moderately correlated [45,64], and the quality of coparental alliance influence children’s perception of coparenting [66]. In addition, because child externalizing problems are overt and obvious, parents and children may have a consistent judgment of child externalizing problems. Thus, father-report and mother-report results did not show significant differences.

With respect to internalizing symptoms, the relation between coparenting and internalizing symptoms was significantly higher among child-report than father-report data, whereas the association was not significantly different between child-report and mother-report data. This result indicates that compared to father-report coparenting, child-report coparenting is a more proximal factor that influences children’s adjustment problems. More attention should be paid to children’s perception of parents’ coparenting alliance.

### 4.4. Moderation of Gender

The results indicate that the girl percentage did not moderate the association between coparenting and children’s externalizing or internalizing problems, indicating that no gender risk exists in the influence of coparenting behavior on children’s adjustment problems. This result is not consistent with previous studies [18,26,60], and did not support the male vulnerability model or the distinct pathways model. That is, the results did not indicate girls’ vulnerability to internalizing problems or boys’ vulnerability to externalizing problems when confronting negative coparenting experiences. Nor did the results support boys’ greater vulnerability to negative coparenting compared to girls. This result is surprising and interesting. However, further evidence is still required; because we did not examine the relationship between coparenting dimensions and child adjustment problems across gender, we may not have been able to find more details about the gender difference.

### 4.5. Limitation and Future Direction

Several limitations in the current study should be addressed. Firstly, we could not put all studies about coparenting into the four dimensions. For instance, we did not distinguish between covert and overt coparenting conflicts [18]. Second, some coparenting behavior is hard to categorize into four dimensions. For example, Metz et al. [10] describe coparenting through four dimensions: competitive, neutral, mutual, and cooperation. Neutral behavior demonstrates that the parents were engaged in the task but were not performing any coparenting initiatives (for example, the parent watches while their partner dresses the child). This is considered a negative attitude, but it is difficult to categorize into the dimension of conflict or C-T. Similarly, Jacobvitz’s measure of coparenting through an enmeshed scale, controlling scale, emotional disengagement scale, hostility scale, and balanced scale was also not included in this meta-analysis either [76]. Third, we did not control for the influence of parental and marital relationships, although many studies showed that marital relationships have a certain influence on coparenting or child adjustment [23,33,36].

This meta-analysis highlights multiple areas for future research. Firstly, most studies included in our meta-analysis only examined the relationship between coparenting behaviors and child adjustment problems and did not emphasize the interaction between father and mother coparenting style. Several studies found that parents perform differently in terms of coparenting behavior [10,77]. Parents can display consistent coparenting behaviors (both parents being supportive or undermining) or divergent coparenting behaviors (only one parent being supportive or undermining). For instance, the association between infant negative affectivity and child anxiety was moderated by parents’ divergent coparenting. Specifically, it was stronger when mothers were cooperative while fathers were neutral, and weaker when fathers were cooperative while mothers were neutral. When fathers step forward (i.e., being cooperative) and mothers step back (i.e., leaving space), they may protect their at-risk child from developing anxiety [10,77]. Future studies should include an investigation of the discrepancy between father coparenting and mother coparenting behavior and pay attention to the difference and interaction between father and mother coparenting.

Secondly, future studies should focus on bidirectional influences between dimensions of coparenting and child adjustment problems. Several studies discovered that risk behavior predicted less shared decision making, and shared decision making protected against increased risk behavior for boys. For girls and boys, parents’ joint involvement predicted fewer risk behaviors, and lower levels of risk behavior predicted higher levels of joint involvement. In contrast, boys’ and girls’ depressive symptoms predicted less joint involvement [28]. Additionally, interparental hostility in Time 1 will lead to later child psychological problems, and later child psychological problems will increase interparental dysphoria [48]. This kind of bidirectional influence plays an important role in intervention.

## Figures and Tables

**Table 1:** Correlations between coparenting and externalizing and internalizing symptoms.

Measure	Coparenting-Externalizing				Coparenting-Internalizing			
	k	r	95% CI	τ^2^	k	r	95% CI	τ^2^
Main average correlation	138	−0.173 ***	[−0.194, −0.151]	0.011	141	−0.162 ***	[−0.180, −0.144]	0.008
Mono-informant bias								
1. YES	30	−0.156 ***	[−0.202, −0.109]	0.012	39	−0.199 ***	[−0.232, −0.167]	0.005
2. NO	106	−0.177 ***	[−0.204, −0.151]	0.011	100	−0.147 ***	[−0.171, −0.123]	0.007
Longitudinal study								
1. YES	65	−0.144 ***	[−0.173, −0.114]	0.008	56	−0.125 ***	[−0.153, −0.097]	0.005
2. NO	71	−0.197 ***	[−0.231, −0.162]	0.014	77	−0.194 ***	[−0.223, −0.166]	0.009
Developmental stage								
1. Toddler	46	−0.134 ***	[−0.177, −0.089]	0.012	41	−0.164 ***	[−0.201, −0.127]	0.004
2. Childhood	58	−0.198 ***	[−0.225, −0.163]	0.009	54	−0.149 ***	[−0.180, −0.117]	0.008
3. Adolescence	23	−0.174 ***	[−0.221, −0.127]	0.009	29	−0.194 ***	[−0.238, −0.281]	0.012
Coparenting data reporter								
1. Father	16	−0.179 ***	[−0.239, −0.118]	0.009	18	−0.117 ***	[−0.172, −0.062]	0.008
2. Mother	26	−0.170 ***	[−0.219, −0.121]	0.011	29	−0.180 ***	[−0.221, −0.138]	0.008
3. Children	25	−0.179 ***	[−0.225, −0.132]	0.011	34	−0.232 ***	[−0.265, −0.198]	0.006
Types of coparenting								
1. Integrity	20	−0.141 *	[−0.252, −0.028]	0.049	19	−0.153 ***	[−0.202, −0.103]	0.000
2. Cooperation	34	−0.111 ***	[−0.153, −0.068]	0.006	30	−0.121 ***	[−0.158, −0.084]	0.003
3. Conflict	67	0.201 ***	[0.231, 0.171]	0.010	74	0.189 ***	[0.215, 0.163]	0.008
4. C-T	15	0.168 ***	[0.221, 0.113]	0.007	16	0.126 ***	[0.158, 0.065]	0.010

Note. *** *p* < 0.001, * *p* < 0.05; k = number of effect sizes; r = point estimate of the effect size; CI = confidence interval; τ^2^ = Between-study sampling variance.

**Table 2:** Moderations on the correlation between coparenting and externalizing and internalizing symptoms.

Correlation	β	SE	z Value	95% CI	*p* Value
Coparenting-Externalizing					
Publication year	0.001	0.002	0.712	[−0.002, 0.004]	0.477
Mono-informant bias					
NO vs. YES	0.023	0.028	0.795	[−0.033, 0.078]	0.427
Research method					
Cross-sectional vs. Longitudinal	**0.054**	**0.024**	**2.203**	**[0.006, 0.102]**	**0.028**
Data reporter					
Father vs. Mother	0.009	0.042	0.227	[−0.072, 0.091]	0.820
Father vs. Children	0.000	0.041	0.000	[−0.080, 0.080]	1.000
Mother vs. Children	−0.010	0.035	−0.270	[−0.078, 0.059]	0.787
Development stage					
Toddler vs. Child	**−0.066**	**0.029**	**−2.300**	**[−0.123, −0.010]**	**0.022**
Toddler vs. Adolescent	−0.043	0.035	−1.236	[−0.111, 0.025]	0.217
Child vs. Adolescent	0.0232	0.032	0.722	[−0.040, 0.086]	0.470
Individualism	−0.001	0.001	−0.553	[−0.002, 0.001]	0.580
Female percentage	0.002	0.001	1.199	[−0.001, 0.004]	0.231
Types of coparenting					
Cooperation vs. Conflict	**−0.092**	**0.030**	**−3.050**	**[−0.150, −0.033]**	**0.002**
Cooperation vs. C-T	−0.061	0.041	−1.476	[−0.142, 0.020]	0.140
Cooperation vs. Integrity	−0.022	0.045	−0.480	[−0.109, 0.066]	0.631
Conflict vs. C-T	0.031	0.037	0.840	[−0.041, 0.102]	0.401
Conflict vs. Integrity	0.070	0.040	1.731	[−0.009, 0.149]	0.083
Integrity vs. C-T	−0.039	0.049	−0.798	[−0.136, 0.057]	0.425
Coparenting-Internalizing					
Publication year	0.000	0.002	−0.285	[−0.003, 0.003]	0.775
Mono-informant bias					
NO vs. YES	**−0.053**	**0.022**	**−2.365**	**[−0.097, −0.009]**	**0.018**
Research method					
Cross-sectional vs. Longitudinal	**0.071**	**0.022**	**3.298**	**[0.029, 0.113]**	**0.001**
Data reporter					
Father vs. Mother	−0.064	0.036	−1.783	[−0.134, 0.006]	0.075
Father vs. Children	**−0.119**	**0.034**	**−3.446**	**[−0.186, −0.051]**	**0.001**
Mother vs. Children	−0.055	0.029	−1.871	[−0.112, 0.003]	0.061
Development stage					
Toddler vs. Child	0.018	0.028	0.623	[−0.038, 0.073]	0.534
Toddler vs. Adolescent	−0.028	0.032	−0.891	[−0.090, 0.034]	0.373
Child vs. Adolescent	−0.046	0.028	−1.637	[−0.100, 0.009]	0.102
Individualism	0.001	0.001	0.707	[−0.001, 0.002]	0.480
Female percentage	0.000	0.001	0.093	[−0.002, 0.002]	0.926
Types of coparenting					
Cooperation vs. Conflict	**−0.069**	**0.027**	**−2.557**	**[−0.121, −0.016]**	**0.011**
Cooperation vs. C-T	−0.005	0.036	−0.139	[−0.076, 0.066]	0.890
Cooperation vs. Integrity	−0.027	0.041	−0.640	[−0.108, 0.055]	0.522
Conflict vs. C-T	**0.064**	**0.031**	**2.043**	**[0.003, 0.125]**	**0.041**
Conflict vs. Integrity	0.042	0.037	1.145	[−0.030, 0.115]	0.252
Integrity vs. C-T	0.021	0.044	0.484	[−0.065, 0.108]	0.629

Note. Several models were run for subgroup comparisons among moderators. For the convenience of presentation, subgroup comparisons within categorical moderators are all listed in the model. CI = confidence interval. The second group in each group comparison variable is the reference group (e.g., in cross-sectional vs. longitudinal, longitudinal is the reference group in the dummy coding of research method). Bold means that the results were significant.

**Table 3:** Coparenting as a predictor of change in child adjustment.

Association	k	r	95%CI
Coparenting-Externalizing	65	−0.144 ***	[−0.173, −0.114]
Integrity	7	−0.129 *	[−0.221, −0.035]
Cooperation	18	−0.076 ***	[−0.115, −0.037]
Conflict	29	0.173 ***	[−0.197, −0.150]
C-T	9	0.122 ***	[−0.161, −0.084]
Coparenting-Internalizing	56	−0.125 ***	[−0.153, −0.197]
Integrity	6	−0.121 *	[−0.220, −0.019]
Cooperation	13	−0.069 **	[−0.116, −0.021]
Conflict	28	0.132 ***	[−0.172, −0.091]
C-T	7	0.135 ***	[−0.176, −0.093]

Note. *** *p* < 0.001, ** *p* < 0.01, * *p* < 0.05; k = number of effect sizes; r = point estimate of the effect size; CI = confidence interval.

## Data Availability

The data presented in this study are available on request from the corresponding author.

## Supplementary Materials

The following supporting information can be downloaded at: https://www.mdpi.com/article/10.3390/ijerph191610346/s1, Table S1: Original studies included in meta-analysis. The references of 93 studies included in this meta-analysis (also listed in the supplementary materials) are [1,2,3,4,5,6,7,9,10,11,16,17,18,19,20,22,23,24,25,26,27,28,29,30,31,32,33,34,35,36,37,38,39,41,42,44,45,46,48,49,50,52,53,54,60,61,62,64,65,67,74,75,77,78,79,80,81,82,83,84,85,86,87,88,89,90,91,92,93,94,95,96,97,98,99,100,101,102,103,104,105,106,107,108,109,110,111,112,113,114,115,116,117].
