# Supplementary material for: The Association between Coparenting Behavior and Internalizing/Externalizing Problems of Children and Adolescents: A Meta-Analysis
Source: Int J Environ Res Public Health. 2022 Aug 19;19(16):10346. doi: 10.3390/ijerph191610346 (PMC9407961; doi:10.3390/ijerph191610346)
Supplement: Supplementary file 1 [file ijerph-19-10346-s001.zip › ijerph-1817319-supplementary.pdf]

Table S1 Original studies included in meta-analysis

Author(year)	Status	Individualism	Longitudinal study	Mono-informant bias	sibling	Children		Coparenting measure	Data reporter	Adjustment assessment	r	n
						Development stage	Female Percentage					
Abidin(1995)a	Pub.	91	0	0		1	56%	Q	M	ESBS	-0.116	237
Abidin(1995)b	Pub.	91	0	0		1	56%	Q	F	ESBS	-0.120	173
Altenburger(2017)	Pub.	91	1	0		1	49%	OB	S	ITSEA	-0.164	114
Atkinson(2009)	Pub.	90	0	1		3	46%	Q	S	CBCL	-0.280	236
Bakhtiari(2017)	Pub.	41	0	1		3		Q	S	other	-0.310	164
Baptista(2018)	Pub.	27	0	0	68.30%	1	47%	Q	F	other	0.440	70
Baril(2007)	Pub.	91	1	0	100%	3		Q	PS	other	-0.158	177
Bearss(1998)	Pub.	91	0	1		2	47%	Q	P	ECBI	-0.575	53
Beckmeyer(2014)	Pub.	91	0	0		2	52%	Q	PS	SSRS	-0.118	270
Benson(2008)	Pub.	91	0	0		2	52%	Q	S	CBCL	-0.238	1893
Bosco(2009)	Pub.	91	0	0		3	50%	Q	S	CBCL	-0.495	150
Bradford(2003)	Pub.	91	0	1		3		Q	S	CBCL	0.060	9050
Bradford(2008)	Pub.	91	0	1		3	52%	Q	S	CBCL	-0.273	641
Breslend(2016)	Pub.	91	0	0				Q		CBCL	-0.176	180
Brock(2015)a	Pub.	91	0	0		2	51%	Mix.	M	CBCL	-0.096	416
Brock(2015)b	Pub.	91	0	0		2	51%	Mix.	C	CBCL	-0.077	416
Brody(1996)	Pub.	91	0	0			53%	OB	C	CDI	-0.130	90
Brody(1999)	Pub.	91	0	0	100%	2	53%	Mix.	PS	other	-0.227	85
Buchanan(1991)	Pub.	91	1	0			49%	ITV	PS	other	-0.033	336
Buehler(2009)a	Pub.	91	1	0		3	51%	Q	S	CBCL	-0.125	366
Buehler(2009)b	Pub.	91	1	0		3	51%	Q	F	CBCL	-0.151	366
Buehler(2009)c	Pub.	91	1	0		3	51%	Q	M	CBCL	-0.106	366

Buehler(2009)a	Pub.	91	1	0		2	51%	Q	M	other	-0.093	414
Buehler(2009)b	Pub.	91	1	0		2	51%	Q	F	other	-0.110	414
Buehler(2016)	Pub.	91	0	1		3	56%	Q	S	CBCL	-0.340	337
Camisasca(2019)	Pub.	76	0	0	78.50%	2	51%	Q	M	CBCL	-0.429	101
Choi(2018)	Pub.	91	1	1		2		ITV	M	CBCL	-0.090	1773
Cummings(2006)a	Pub.	91	1	0		2	50%	Q	M	CBCL	-0.141	226
Cummings(2006)b	Pub.	91	1	0		2	50%	Q	F	CBCL	-0.199	226
Cummings(2006)c	Pub.	91	1	0		2	50%	Q	S	CBCL	-0.138	226
Cummings(2015)a	Pub.	91	1	0		3	50%	Q	M	other	-0.112	280
Cummings(2015)b	Pub.	91	1	0		3	50%	Q	F	other	-0.123	280
Cummings(2015)c	Pub.	91	1	0		3	50%	Q	S	other	-0.134	280
Davies(2004)	Pub.	91	1	0		2	56%	Q	P	CBCL	-0.144	221
Davies(2007)	Pub.	91	1	0		2	55%	Q	PS	CBCL	-0.105	178
Davies(2012)	Pub.	91	0	0		3	52%	Q	P	CBCL	-0.145	250
Davies(2016）	Pub.	91	1	0		2	55%	OB	C	CBCL	-0.040	232
Davis(2009)a	Pub.	91		0		2	53%	Q	S	CDI	-0.350	153
Davis(2009)b	Pub.	91		0		2	53%	Q	M	CDI	-0.095	153
Deal(1989)a	Pub.	91	0	0		1	51%	Q	M	BPC	-0.030	136
Deal(1989)b	Pub.	91	0	0		1	51%	Q	F	BPC	-0.380	136
Etkin(2014)	Pub.	91	1	0		3	41%	Q	S	CBCL	-0.098	301
Farr(2013)	Pub.	91	0	0	50%	1	52%	OB	C	CBCL	-0.076	50
Fear(2009)	Pub.	91	0	1		2	54%	Q	Y	CBCL	-0.230	108
Feinberg(2007)a	Pub.	91	1	0		3	48%	Q	M	other	-0.241	259
Feinberg(2007)b	Pub.	91	1	0		3	48%	Q	F	other	-0.208	259
Feinberg(2007)b	Pub.	91	1	0		3	48%	Q	F	other	-0.208	259
Floyd(1991)	Pub.	91	0	0		2	65%	Q	PS	CBCL	-0.370	32
Forehand(2003)	Pub.	91	1	1		2	49%	Q	M	CBCL	-0.155	117

Fosco(2008)	Pub.	91	0	0		2	49%	Q	Mix	CBCL	-0.268	150
Franck(2007)	Pub.	91	0	0		2	51%	Q	S	CBCL	-0.160	416
Gallegos(2017)	Pub.	91	0	0		1	41%	OB	C	other	-0.310	108
Gagne(2007)	Pub.	80	0	1		2	58%	Q	S	CBCL	-0.151	136
Gomulak(2006)	Pub.	91	0	0		2	55%	Mix.		CBCL	-0.150	227
Groenendyk(2007)a	Pub.	91	0	1		1	52%	Mix.	M	other	-0.305	106
Groenendyk(2007)b	Pub.	91	0	1		1	52%	Mix.	F	other	-0.151	106
Grych(2004)	Pub.	91	0	1		1	60%	Q	S	CBCL	-0.235	338
Hentges(2015)	Pub.	91	1			1	44%	Mix.	Mix	CBCL	-0.231	201
Huang(2019)	Pub.	20	0	0	41.20%	3	54%	Q	F	other	-0.002	3045
Jennifer(2019)a	Pub.	91	1	1		2	0%	Q	M	CBCL	-0.326	259
Jennifer(2019)b	Pub.	91	1	1		2	100%	Q	M	CBCL	-0.150	259
Jia(2012)	Pub.	91	1	0		1	54%	OB	C	CBCL	0.016	79
Johnson(1999)	Pub.	91	1	0		2	40%	Mix.	C	CABI	-0.240	63
Jones(2005)	Pub.	91	1	1		3	50%	Q	M	CBCL	-0.118	277
Jouriles(1991)	Pub.	91	0	1		1		ITV	M	CBCL	-0.222	85
Jouriles(2014)	Pub.	91	1	0		2	42%	Q	S	CBCL	-0.080	119
Katz(2004)	Pub.	91	0	0		1	38%	OB	C	CBCL	-0.146	130
Keeports(2017)	Pub.	91	0	1		3	58%	Q	S	other	-0.280	255
Kim(2008)	Pub.	91	0	1			47%	Q	S	BASC	-0.225	169
King(1995)	Pub.	91				3	63%	Q	M	other	-0.355	68
Kolak(2008)	Pub.	91	0	0	56%	1	46%	OB	C	other	-0.148	104
Kolak(2013)	Pub.	91	1	0	100%	1	54%	OB	C	CBCL	-0.015	209
Kouros(2010)	Pub.	91	1	0		3	55%	Q	PS	CBCL	-0.180	235
Lamela(2016)	Pub.	27	0	1		2		Q	PS	SDQ	-0.177	314
Larsen(2007)	Pub.	80	1			3	52%	Q	S	other	-0.120	932
Latham(2017)a	Pub.	89	1	1	100%	1	50%	Q	M	ECBI	-0.055	212
Latham(2017)b	Pub.	80	1	1	100%	1	50%	Q	F	ECBI	-0.185	212

Lee(2005)a	Pub.	91	0	0	50%	1		Q	F	CBCL	-0.176	122
Lee(2005)b	Pub.	80	0	0	50%	1		Q	M	CBCL	-0.313	122
Lindahl(1999)a	Pub.	91	0	0		2	0%	Mix.	C	CBCL	-0.615	113
Lindahl(1999)b	Pub.	91	0	0		2	0%	Mix.	M	CBCL	-0.290	113
Lindahl(1999)c	Pub.	91	0	0		2	0%	Mix.	F	CBCL	-0.385	113
Liu(2016)	Pub.	20	0	1		3	46%	Q	S	other	-0.150	1407
Liu(2018)	Un-Pub.	20	0	1		1	47%	Q	P		-0.224	432
Mack(2018)a	Pub.	91	0	1		1	74%	Q	F	CBCL	-0.080	135
Mack(2018)b	Pub.	91	0	1		1	74%	Q	M	CBCL	-0.187	135
Mahoney(1997)a	Pub.	91	0	0		2	23%	Q	M	CBCL	-0.115	146
Mahoney(1997)b	Pub.	91	0	0		2	23%	Q	F	CBCL	-0.187	146
McConnell(2002)a	Pub.	80	0	0		2	0%	Mix.	C	CBCL	-0.121	67
McConnell(2002)b	Pub.	80	0	0		2	100%	Mix.	C	CBCL	-0.010	67
McDonald(2006)	Pub.	91	0	0		2	51%	Q	S	Mix	-0.263	179
McHale(1998)a	Pub.	91	1	0		1	59%	OB	C	Mix	-0.235	37
McHale(1998)b	Pub.	91	1	0		1	59%	OB	F	Mix	-0.223	37
McHale(1998)c	Pub.	91	1	0		1	59%	OB	M	Mix	-0.252	37
McHale(1999)a	Pub.	91	0	0		1	0%	OB	C		-0.276	21
McHale(1999)b	Pub.	91	0	0		1	100%	OB	C		0.015	21
McHale(2000)	Pub.	20	0	1	5%	1	36%	Q	PS	CABI	-0.162	100
Metz(2017)	Pub.	91	1	0		1	59%	OB	C	PAS	-0.010	102
Mueller(2015)	Pub.	91		0		2	49%	Q	S	RCMAS	-0.191	531
Murphy(2016)	Pub.	91	1	0		2	44%	OB	C	CBCL	-0.262	71
O'Donnell(2010)	Pub.	91	1	1		2	59%	Q	S	CDI	-0.350	88
O'Leary(2005)a	Pub.	91	0	0		1	0%	Q	F	CBCL	-0.245	104
O'Leary(2005)b	Pub.	91	0	0		1	100%	Q	F	CBCL	-0.138	104
O'Leary(2005)c	Pub.	91	0	0		1	0%	Q	M	CBCL	-0.318	104
O'Leary(2005)d	Pub.	91	0	0		1	100%	Q	M	CBCL	-0.205	104

Rabinowitz(2016)	Pub.	91	1	1	2	31%	Q	M	CBCL	-0.338	775
Riina(2014)a	Pub.	91	1	1	2	0%	ITV	Mix	other	-0.038	203
Riina(2014)b	Pub.	91	1	1	2	100%	ITV	Mix	other	0.030	203
Rosenfield(2014)	Pub.	91		0	2	49%	Q	S	Mix	-0.194	539
Schoppe(2001)	Pub.	91	1	0	1		OB	C	CBCL	-0.396	46
Schoppe(2009)	Pub.	91	1	0	1	48%	OB	C	Mix	-0.058	92
Schrodt(2013)	Pub.	91		1	2	59%	Q	S	other	-0.200	364
Schrodt(2013)	Pub.	91		1		59%	Q	S	other	-0.125	129
Schrodt(2018)a	Pub.	91	0	0	3	79%	Q	M	other	-0.225	225
Schrodt(2018)b	Pub.	91	0	0	3	79%	Q	F	other	-0.141	225
Scrimgeour(2013)	Pub.	91	1	0	1	60%	OB	C	HBO	-0.230	58
Shelton(2008)	Pub.	89	1	0	2	49%	Q	PS	CBCL	-0.109	352
Snyder(1988)	Pub.	91	0	0		48%	Q	PS	PIC	-0.317	110
Stright(2003)	Pub.	91	0	0	2	29%	Mix.	C	CBCL	-0.380	52
Umemura(2015)	Pub.	91	1	0	2		OB	C	CBCL	-0.163	85
Vaughn(1988)a	Pub.	91	1	0	3	0%	Q	PS	DPQ	-0.241	35
Vaughn(1988)b	Pub.	91	1	0	3	100%	Q	PS	DPQ	-0.094	33
Wang(2001)a	Pub.	91	0	0	2	35%	Q	F	other	-0.350	120
Wang(2001)b	Pub.	91	0	0	2	35%	Q	M	other	-0.040	120
Yan(2018)	Pub.	91	1	1	1	49%	Q	M	ITSEA	-0.220	182
Ying(2018)	Pub.	20	0		2	45%	Q	S	CES	-0.350	437
Zhao.T(2017)a	Un-Pub.	20	0	0		51%	Q	F	other	-0.212	376
Zhao.T(2017)b	Un-Pub.	20	0	0		51%	Q	M	other	-0.234	376

Note. Longitudinal study and Mono-informant bias (1 = Yes; 0 = No); Development stage (1 = Toddler; 2 = Child; 3 = Adolescent); Research Method (Q = Questionnaire; OB = Observation; ITV = Interview; Mix. = two of these methods were used); Data reporter (C = Coder; F = Father; M = Mother; S= self-report; PS = parents-report; P = parent-report); Adjustment assessment (CBCL = Child Behavior Checklist; ITSEA = Infant Toddler Social Emotional Assessment; BPC = Behavior Problem CheckList; CABI = Child Adaptive Behavior Inventory; CDI = Children's Depression Inventory; CES = Centre for Epidemiologic Studies; DPQ = Differenbal Personality Questionnaire; ECBI = Eyberg Child

Behavior Inventory; HBO = the Mac Arthur Health and Behavior Questionnaire; PAS = Preschool Anxiety Scale; BASC = Behavior Assessment System for Children; PIC = Personality Inventory for Children; RCMAS = Revised Children's Manifest Anxiety Scale; SDQ = Strengths and Difficulties Questionnaire; SSRS = Social Skills Rating System)

References: List of 93 articles used in the meta-analysis

*Abidin, R. R., & Brunner, J. F. (1995). Development of a Parenting Alliance Inventory. *Journal of Clinical Child Psychology*. *24(1),* 31–40. doi:10.1207/s15374424jccp2401_4

*Altenburger, L. E., Lang, S. N., Schoppe-Sullivan, S. J., Dush, C. M. K., & Johnson, S. (2015). Toddlers' differential susceptibility to the effects of coparenting on social-emotional adjustment. *International Journal of Behavioral Development, 41*(2), 228–237. doi:10.1177/0165025415620058

*Atkinson, E. R., Dadds, M. R., Chipuer, H., & Dawe, S. (2009). Threat is a multidimensional construct: Exploring the role of children's threat appraisals in the relationship between interparental conflict and child adjustment. *Journal of Abnormal Child Psychology, 37*(2), 281–292. doi:10.1007/s10802-008-9275-z

*Bakhtiari, F., Plunkett, S. W., & Alpizar, D. (2017). Family qualities, self-deprecation, and depressive symptoms of Zoroastrian young adults in immigrant families. *Journal of Immigrant and Minority Health, 19*(3), 645–654. doi:10.1007/s10903-016-0476-1

*Baptista, J., Sousa, D., Soares, I., & Martins, C. (2018). Fathers' sensitive guidance moderates the association between coparenting and behavioral regulation in preschoolers. *International Journal of Behavioral Development, 42(6)*, 574–580. doi:10.1177/0165025418761816

*Baril, M. E., Crouter, A. C., & McHale, S. M. (2007). Processes linking adolescent well-being, marital love, and coparenting. *Journal of Family Psychology, 21*(4), 645–654. doi:10.1037/0893-3200.21.4.645

*Bearss, K. E., & Eyberg, S. (1998). A test of the Parenting Alliance Theory. *Early Education & Development, 9*(2)*,* 179–185. doi:10.1207/s15566935eed0902_5

*Beckmeyer, J. J., Coleman, M., & Ganong, L. H. (2014). Postdivorce coparenting typologies and children's adjustment. *Family Relations, 63*(4), 526–537. doi:10.1111/fare.12086

*Benson, M. J., Buehler, C., & Gerard, J. M. (2008). Interparental hostility and early adolescent problem behavior - Spillover via maternal acceptance, harshness, inconsistency, and intrusiveness. *Journal of Early Adolescence, 28*(3), 428–454. doi:10.1177/0272431608316602

*Bosco, G. L., Renk, K., Dinger, T. M., Epstein, M. K., & Phares, V. (2003). The connections between adolescents' perceptions of parents, parental psychological symptoms, and adolescent functioning. *Journal of Applied Developmental Psychology, 24*(2), 179–200. doi:10.1016/s0193-

3973(03)00044-3

*Bradford, K., Barber, B. K., Olsen, J. A., Maughan, S. L., Erickson, L. D., Ward, D., & Stolz, H. E. (2003). A multi-national study of interparental conflict, parenting, and adolescent functioning. *Marriage & Family Review, 35*(3-4), 107–137. doi:10.1300/J002v35n03_07

*Bradford, K., Vaughn, L. B., & Barber, B. K. (2008). When there is conflict - Interparental conflict, parent-child conflict, and youth problem behaviors. *Journal of Family Issues, 29*(6), 780–805. doi:10.1177/0192513x07308043

*Breslend, N. L., Parent, J., Forehand, R., Compas, B. E., Thigpen, J. C., & Hardcastle, E. (2016). Parental depressive symptoms and youth internalizing and externalizing problems: The moderating role of interparental conflict. *Journal of Family Violence, 31*(7), 823–831. doi:10.1007/s10896-016-9817-z

*Brock, R. L., & Kochanska, G. (2015). Decline in the quality of family Relationships predicts escalation in children's internalizing symptoms from middle to late Childhood. *Journal of Abnormal Child Psychology, 43*(7), 1295–1308. doi:10.1007/s10802-015-0008-9

*Brody, G. H., & Flor, D. L. (1996). Coparenting, family interactions, and competence among African American youths. *New Directions for Child Development*, 74, 77–91. doi: 10.1002/cd.23219967407

*Brody, G. H., Stoneman, Z., Smith, T., & Gibson, N. M. (1999). Sibling relationships in rural African American families. *Journal of Marriage and Family*, *63*(4), 996–1008. doi:10.1111/j.1741-3737.2001.00996.x

*Buchanan, Christy M.，Maccoby, Eleanor E., & Dornbusch, Sanford M.(1991). Caught between parents adolescents' experience in divorced home. *Child Development, 62*(5), 1008-1029. URL: http://www.jstor.org/stable/1131149

*Buehler, C., Franck, K. L., & Cook, E. C. (2009). Adolescents' triangulation in marital conflict and peer relations. *Journal of Research on Adolescence, 19*(4), 669–689. doi:10.1111/j.1532-7795.2009.00616.x

*Buehler, C., Krishnakumar, A., Stone, G., Anthony, C., Pemberton, S., Gerard, J., & Barber, B. K. (2016). Interparental conflict styles and youth problem behaviors: A two-sample replication study. *Journal of Marriage and Family*, *60*(1), 119–132.

*Buehler, C., & Welsh, D. P. (2009). A process model of adolescents' triangulation into parents' marital conflict: The role of emotional reactivity. *Journal of Family Psychology, 23*(2), 167–180. doi:10.1037/a0014976

*Camisasca, E., Miragoli, S., Di Blasio, P., & Feinberg, M. (2019). Co-parenting mediates the influence of marital satisfaction on child adjustment: The conditional indirect effect by parental empathy. *Journal of Child and Family Studies, 28*(2), 519–530. doi:10.1007/s10826-018-1271-5

*Choi, J.-K., & Becher, E. H. (2018). Supportive coparenting, parenting stress, harsh parenting, and child behavior problems in nonmarital families. *Family Process*, *58*(2), 404–417. doi:10.1111/famp.12373

*Cummings, E. M., Koss, K. J., & Davies, P. T. (2015). Prospective relations between family conflict and adolescent maladjustment: Security in

the family system as a mediating process. *Journal of Abnormal Child Psychology, 43*(3), 503–515. doi:10.1007/s10802-014-9926-1

*Cummings, E. M., Schermerhorn, A. C., Davies, P. T., Goeke-Morey, M. C., & Cummings, J. S. (2006). Interparental discord and child adjustment: Prospective investigations of emotional security as an explanatory mechanism. *Child Development, 77*(1), 132–152. doi:10.1111/j.1467-8624.2006.00861.x

*Davies, P. T., Hentges, R. F., Coe, J. L., Martin, M. J., Sturge-Apple, M. L., & Cummings, E. M. (2016). The multiple faces of interparental conflict: Implications for cascades of children's insecurity and externalizing problems. *Journal of Abnormal Psychology, 125*(5), 664–678. doi:10.1037/abn0000170

*Davies, P. T., Sturge-Apple, M. L., Cicchetti, D., & Cummings, E. M. (2007). The role of child adrenocortical functioning in pathways between interparental conflict and child maladjustment. *Developmental Psychology, 43*(4), 918–930. doi:10.1037/0012-1649.43.4.918

*Davies, P. T., Sturge-Apple, M. L., Cicchetti, D., Manning, L. G., & Vonhold, S. E. (2012). Pathways and processes of risk in associations among maternal antisocial personality symptoms, interparental aggression, and preschooler's psychopathology. *Development and Psychopathology, 24*(3), 807–832. doi:10.1017/s0954579412000387

*Davis, K. A., & Epkins, C. C. (2009). Do private religious practices moderate the relation between family conflict and preadolescents' depression and anxiety symptoms? *Journal of Early Adolescence, 29*(5), 693–717. doi:10.1177/0272431608325503

*Deal, J. E., Halverson, C. F., & Wampler, J. a. K. S. (1989). Parental agreement on child-rearing orientations: relations to parental, maiital, family, and child characteristics. *Child Development*, *60*(5), 1025–34. doi: 10.1111/j.1467-8624.1989.tb03533.x

*Etkin, R. G., Koss, K. J., Cummings, E. M., & Davies, P. T. (2014). The differential impact of parental warmth on externalizing problems among triangulated adolescents. *Journal of Genetic Psychology, 175*(2), 118–133. doi:10.1080/00221325.2013.813437

*Farr, R. H., & Patterson, C. J. (2013). Coparenting among lesbian, gay, and heterosexual couples: Associations with adopted children's outcomes. *Child Development, 84*(4), 1226–1240. doi:10.1111/cdev.12046

*Fear, J. M., Champion, J. E., Reeslund, K. L., Forehand, R., Colletti, C., Roberts, L., & Compas, B. E. (2009). Parental depression and interparental conflict: Children and adolescents' self-blame and coping responses. *Journal of Family Psychology, 23*(5), 762–766. doi:10.1037/a0016381

*Feinberg, M. E., Kan, M. L., & Hetherington, E. M. (2007). The longitudinal influence of coparenting conflict on parental negativity and adolescent maladjustment. *Journal of Marriage and Family, 69*(3), 687–702. doi:10.1111/j.1741-3737.2007.00400.x

*Floyd, F. J., & Zmich, D. E. (1991). Marriage and the parenting partnership: Perceptions and interactions of parents with mentally retarded and typically developing children. *Child Development*, *62*(6), 1434–1448. doi:10.111/j.1467-8624. 1991. tb01616.x

*Forehand, R., & Jones, D. J. (2003). Neighborhood violence and coparent conflict: Interactiveinfluence on child psychosocial adjustment. *Journal*

of Abnormal Child Psychology, *31*(6):591–604. doi:10.1023/a:1026206122470

*Fosco, G. M., & Grych, J. H. (2008). Emotional, cognitive, and family systems mediators of children's adjustment to interparental conflict. *Journal of Family Psychology, 22*(6), 843–854. doi:10.1037/a0013809

*Franck, K. L., & Buehler, C. (2007). A family process model of marital hostility, parental depressive affect, and early adolescent problem behavior: The roles of triangulation and parental warmth. *Journal of Family Psychology, 21*(4), 614–625. doi:10.1037/0893-3200.21.4.614

*Gagne, M. H., Drapeau, S., Melancon, C., Saint-Jacques, M. C., & Lepine, R. (2007). Links between parental psychological violence, other family disturbances, and children's adjustment. *Family Process, 46*(4), 523–542. doi:10.1111/j.1545-5300.2007.00230.x

*Gallegos, M. I., Murphy, S. E., Benner, A. D., Jacobvitz, D. B., & Hazen, N. L. (2017). Marital, parental, and whole-family predictors of toddlers' emotion regulation: the role of parental emotional withdrawal. *Journal of Family Psychology, 31*(3), 294–303. doi:10.1037/fam0000245

*Gomulak-Cavicchio, B. M., Davies, P. T., & Cummings, E. M. (2006). The role of maternal communication patterns about interparental disputes in associations between interparental conflict and child psychological maladjustment. *Journal of Abnormal Child Psychology, 34*(6), 757–771. doi:10.1007/s10802-006-9050-y

*Groenendyk, A. E., & Volling, B. L. (2007). Coparenting and early conscience development in the family. *Journal of Genetic Psychology, 168*(2), 201–224. doi:10.3200/gntp.168.2.201-224

*Grych, J. H., Raynor, S. R., & Fosco, G. M. (2004). Family processes that shape the impact of interparental conflict on adolescents. *Development and Psychopathology*, *16*(3), 649–65.doi:10.1017/s0954579404004717

*Hentges, R. F., Davies, P. T., & Cicchetti, D. (2015). Temperament and interparental conflict: the role of negative emotionality in predicting child behavioral problems. *Child Development, 86*(5), 1333–1350. doi:10.1111/cdev.12389

*Huang, B. B., Zou, S. Q., Wu, X. C., & Liu, C. (2019). The association between father coparenting behavior and adolescents'peer attachment: The mediating role of father-child attachment and the moderating role of adolescents'neuroticism. *Psychological Development and Education*, *35*(2),176–183. doi: 10.16187 /j.cnki.issn1001–4918.2019.02.06

*Jia, R., Kotila, L. E., & Schoppe-Sullivan, S. J. (2012). Transactional relations between father involvement and preschoolers' socioemotional adjustment. *Journal of Family Psychology, 26*(6), 848–857. doi:10.1037/a0030245

*Johnson, V. K., Cowan, P. A., & Cowan, C. P. (1999). Children's classroom behavior:the unique contribution of family organization. *Journal of Family Psychology*, *13*(3), 355–371.doi:10.1037/0893-3200.13.3.355

*Jones, D. J., Forehand, R., Dorsey, S., Foster, S., & Brody, G. (2005). Coparent support and conflict in African American single mother-headed families: Associations with maternal and child psychosocial functioning. *Journal of Family Violence, 20*(3), 141–150. doi:10.1007/s10896-005-3650-0

*Jouriles, E. N., Murphy, C. M., Farris, A. M., Smith, D. A., Richters, J. E., & Waters, E. (1991). Marital adjustment, parental disagreements about child rearing, and behavior problems in boys: Increasing the Specificity of the marital assessment. *Child Development*, *62*(6), 1424–1433. doi:10.1111/j.1467-8624. 1991. tb01615.x

*Jouriles, E. N., Rosenfield, D., McDonald, R., & Mueller, V. (2014). Child involvement in interparental conflict and child adjustment problems: a longitudinal study of violent families. *Journal of Abnormal Child Psychology, 42*(5), 693–704. doi:10.1007/s10802-013-9821-1

*Katz, L. F., & Low, S. M. (2004). Marital violence, co-parenting, and family-level processes in relation to children's adjustment. *Journal of family psychology : JFP : journal of the Division of Family Psychology of the American Psychological Association (Division 43), 18*(2), 372–382. doi:10.1037/0893-3200.18.2.372

*Keeports, C. R., & Pittman, L. D. (2017). I wish my parents would stop arguing! the impact of interparental conflict on young adults. *Journal of Family Issues, 38*(6), 839–857. doi:10.1177/0192513x15613821

*Kim, K. L., Jackson, Y., Conrad, S. M., & Hunter, H. L. (2008). Adolescent report of interparental conflict: The role of threat and self-blame appraisal on adaptive outcome. *Journal of Child and Family Studies, 17*(5), 735–751. doi:10.1007/s10826-007-9187-5

*King, C. A., W., N. M., & Segal, H. G. (1995). Parents' marital functioning and adolescent psychopathology. *Journal of Consulting and Clinical Psychology*, *63*(5):749–53, doi:10.1037//0022-006x.63.5.749

*Kolak, A. M., & Vernon-Feagans, L. (2008). Family-level coparenting processes and child gender as moderators of family stress and toddler adjustment. *Infant and Child Development, 17*(6), 617–638. doi:10.1002/icd.577

*Kolak, A. M., & Volling, B. L. (2013). Coparenting moderates the association between firstborn children's temperament and problem behavior across the transition to siblinghood. *Journal of Family Psychology, 27*(3), 355–364. doi:10.1037/a0032864

*Kouros, C. D., Cummings, E. M., & Davies, P. T. (2010). Early trajectories of interparental conflict and externalizing problems as predictors of social competence in preadolescence. *Development and Psychopathology, 22*(3), 527–537. doi:10.1017/s0954579410000258

*Lamela, D., Figueiredo, B., Bastos, A., & Feinberg, M. (2015). Typologies of post-divorce coparenting and parental well-being, parenting quality and children's psychological adjustment. *Child Psychiatry & Human Development, 47*(5), 716–728. doi:10.1007/s10578-015-0604-5

*Larsen, H., Branje, S. J. T., van der Valk, I., & Meeus, W. H. J. (2007). Friendship quality as a moderator between perception of interparental conflicts and maladjustment in adolescence. *International Journal of Behavioral Development, 31*(6), 549–558. doi:10.1177/0165025407080578

*Latham, R. M., Mark, K. M., & Oliver, B. R. (2017). A harsh parenting team? Maternal reports of coparenting and coercive parenting interact in association with children's disruptive behaviour. *Journal of Child Psychology and Psychiatry, 58*(5), 603–611. doi:10.1111/jcpp.12665

*Lee, C. M., Beauregard, C., & Bax, K. A. (2005). Child-related disagreements, verbal aggression, and children's internalizing and externalizing

behavior problems. *Journal of Family Psychology: Journal of the Division of Family Psychology of the American Psychological Association (Division 43), 19*(2), 237–245. doi:10.1037/0893-3200.19.2.237

*Lindahl, K. M., & Malik, N. M. (1999). Marital conflict, family processes, and boys' externalizing behavior in Hispanic American and European American families. *J Clin Child Psychol, 28*(1), 12–24. doi:10.1207/s15374424jccp2801_2

*Liu, D. D.(2018). A study on the relationship between parental marital quality, co-parenting and adolescent problem behavior. *Thesis for Master's degree, Shanxi University.*

*Liu, S., Yu, C. F., Zhen, S. J., Zhang, W., Su, P., & Xu, Y. (2016). Influence of inter-parental conflict on adolescent delinquency via school connectedness: Is impulsivity a vulnerability or plasticity factor? *Journal of Adolescence, 52*, 12–21. doi:10.1016/j.adolescence.2016.07.001

*Mack, R. A., & Gee, C. B. (2018). African American and Latina adolescent mothers' and their children's fathers' reports of coparenting and child behavior problems: Child gender as a moderator. *Journal of Child and Family Studies, 27*(8), 2507–2518. doi:10.1007/s10826-018-1103-7

*Mahoney, A., Jouriles, E. N., & Scavone, J. (1997). Marital adjustment, marital discord over childrearing, and child behavior problems: moderating effects of child age. *Journal of Clinical Child Psychology, 26*(4), 415–423. doi:10.1207/s15374424jccp2604_10

*Marchand-Reilly, J. F., & Yaure, R. G. (2019). The role of parents' relationship quality in children's behavior problems. *Journal of Child and Family Studies, 28*(8), 2199–2208. doi: 10.1007/s10826-019-01436-2

*McConnell, M. C., & Kerig, P. K. (2002). Assessing coparenting in families of school-age children: Validation of the coparenting and family rating system. *Canadian Journal of Behavioural Science/Revue canadienne des sciences du comportement, 34*(1), 44–58. doi:10.1037/h0087154

*McDonald, R., & Grych, J. H. (2006). Young children's appraisals of interparental conflict: Measurement and links with adjustment problems. *Journal of Family Psychology, 20*(1), 88–99. doi:10.1037/0893-3200.20.1.88

*McHale, J., Nirmala, R., & Krasnow, A. D. (2000). Constructing family climates: Chinese mothers' reports of their coparenting behaviour and preschoolers' adaptation. *International Journal of Behavioral Development*, *24*(1), 111–118. doi:10.1080/016502500383548

*McHale, J., & Rasmussen, H. F. (1998). Coparental and family group-level dynamics during infancy: Early family precursors of child and family functioning during preschool. *Development and Psychopathology*, *10*(1), 39–59. doi:10.1017/S0954579498001527

*McHale, J. P., Johnson, D., & Sinclair, R. (1999). Family dynamics, preschoolers' family representations, and preschool peer relationships. *Early Education & Development, 10*(3), 373–401. doi:10.1207/s15566935eed1003_8

*Metz, M., Colonnesi, C., Majdandzic, M., & Bogels, S. M. (2017). When father steps forward and mother steps back: The moderating role of

simultaneity inparents' coparenting behaviors in the development of anxiety in 4-to 30-month-olds. *Infancy, 23*(1), 103–123. doi:10.1111/infa.12199

*Mueller, V., Jouriles, E. N., McDonald, R., & Rosenfield, D. (2015). Children's appraisals and involvement in interparental conflict: Do they contribute independently to child adjustment? *Journal of Abnormal Child Psychology, 43*(6), 1041–1054. doi:10.1007/s10802-014-9953-y

*Murphy, S. E., Jacobvitz, D. B., & Hazen, N. L. (2015). What's so bad about competitive coparenting? family-level predictors of children's externalizing symptoms. *Journal of Child and Family Studies, 25*(5), 1684–1690. doi:10.1007/s10826-015-0321-5

*O'Donnell, E. H., Moreau, M., Cardemil, E. V., & Pollastri, A. (2010). Interparental conflict, parenting, and childhood depression in a diverse urban population: The role of general cognitive style. *Journal of Youth and Adolescence, 39*(1), 12–22. doi:10.1007/s10964-008-9357-9

*O'Leary, S. G., & Vidair, H. B. (2005). Marital adjustment, child-rearing disagreements, and overreactive parenting: predicting child behavior problems. *Journal of Family Psychology, 19*(2), 208–216. doi:10.1037/0893-3200.19.2.208

*Rabinowitz, J. A., Drabick, D. A. G., & Reynolds, M. D. (2016). Family conflict moderates the relation between negative mood and youth internalizing and externalizing symptoms. *Journal of Child and Family Studies, 25*(12), 3574–3583. doi:10.1007/s10826-016-0501-y

*Riina, E. M., & McHale, S. M. (2013). Bidirectional influences between dimensions of coparenting and adolescent adjustment. *Journal of Youth*

and Adolescence, 43(2), 257–269. doi:10.1007/s10964-013-9940-6

*Rosenfield, D., Jouriles, E. N., McDonald, R., & Mueller, V. (2014). Interparental conflict, community violence, and child problems: Making sense of counterintuitive findings. *American Journal of Orthopsychiatry, 84*(3), 275–283. doi:10.1037/h0099805

*Schoppe, S. J., Mangelsdorf, S. C., & Frosch, C. A. (2001). Coparenting, family process, and family structure: Implications for preschoolers' externalizing behavior problems. *Journal of Family Psychology: Journal of the Division of Family Psychology of the American Psychological Association (Division 43), 15*(3), 526–545. doi:10.1037/0893-3200.15.3.526

*Schoppe-Sullivan, S. J., Weldon, A. H., Claire Cook, J., Davis, E. F., & Buckley, C. K. (2009). Coparenting behavior moderates longitudinal relations between effortful control and preschool children's externalizing behavior. *Journal of Child Psychology and Psychiatry, 50*(6), 698–706. doi:10.1111/j.1469-7610.2008.02009.x

*Schrodt, P., & Afifi, T. D. (2018). Negative disclosures and feeling caught mediate coparental communication and mental health. *Personal Relationships, 25*(4), 480–496. doi:10.1111/pere.12256

*Schrodt, P., & Shimkowski, J. R. (2013). Feeling caught as a mediator of co-parental communication and young adult children's mental health and relational satisfaction with parents. *Journal of Social and Personal Relationships, 30*(8), 977–999. doi:10.1177/0265407513479213

*Scrimgeour, M. B., Blandon, A. Y., Stifter, C. A., & Buss, K. A. (2013). Cooperative coparenting moderates the association between parenting

practices and children's prosocial behavior. *Journal of Family Psychology, 27*(3), 506–511. doi:10.1037/a0032893

*Shelton, K. H., & Harold, G. T. (2008). Interparental conflict, negative parenting, and children's adjustment: Bridging links between parents' depression and children's psychological distress. *Journal of Family Psychology, 22*(5), 712–724. doi:10.1037/a0013515

*Snyder, D. K., Klein, M. A., Gdowski, C. L., Faulstich, C., & LaCombe, J. (1988). Generalized dysfunction in clinic and nonclinic families a comparative analysis. *Journal of Abnormal Child Psychology*, *16*(1), 97–109. doi: 10.1007/2FBF00910504

*Stright, A. D., & Neitzel, C. (2003). Beyond parenting: Coparenting and children's classroom adjustment. *International Journal of Behavioral Development*, *27*(1), 31–40. doi:10.1080/01650250143000580

*Umemura, T., Christopher, C., Mann, T., Jacobvitz, D., & Hazen, N. (2015). Coparenting problems with toddlers predict children's symptoms of psychological problems at age 7. *Child Psychiatry & Human Development, 46*(6), 981–996. doi:10.1007/s10578-015-0536-0

*Vaughn, B. E., Block, J. H., & Jack, B. (1988). Parental agreement on child rearing during early childhood and the psychological characteristics of adolescents. *Child Development*, *59*(4), 1020–33.doi:10.1111/j.1467-8624.1988.tb03254.x

*Wang, L., & Crane, D. R. (2001). The relationship between marital satisfaction, marital stability, nuclear family triangulation, and childhood depression. *The American Journal of Family Therapy, 29*(4), 337–347. doi:10.1080/01926180126502

*Yan, J., Schoppe-Sullivan, S. J., & Dush, C. M. K. (2018). Maternal coparenting attitudes and toddler adjustment: Moderated mediation through

father's positive engagement. *Parenting-Science and Practice, 18*(2), 67–85. doi:10.1080/15295192.2018.1444130

*Ying, L. H., Zhou, H. Y., Yu, S.S., Chen, C. S., Jia, X. J., Wang, Y. L., & Lin, C. D. (2018). Parent-child communication and self-esteem mediate the relationship between interparental conflict and children's depressive symptoms. *Child Care Health and Development, 44*(6), 908–915. doi: 10.1111/cch.12610

*Zhao, T. (2017). Research on the relationship between parental work stress, coparenting and junior middle school students' problem behavior. *Thesis for Master' s degree, Shanxi University.*
